# Breast Milk Prefusion F Immunoglobulin G as a Correlate of Protection Against Respiratory Syncytial Virus Acute Respiratory Illness

**DOI:** 10.1093/infdis/jiy477

**Published:** 2018-08-10

**Authors:** Natalie I Mazur, Nicole M Horsley, Janet A Englund, Maaike Nederend, Amalia Magaret, Azad Kumar, Shamir R Jacobino, Cornelis A M de Haan, Subarna K Khatry, Steven C LeClerq, Mark C Steinhoff, James M Tielsch, Joanne Katz, Barney S Graham, Louis J Bont, Jeanette H W Leusen, Helen Y Chu

**Affiliations:** 1 Department of Pediatric Infectious Diseases and Immunology, Wilhelmina Children’s Hospital, Utrecht, The Netherlands; 2 Department of Medicine, University of Washington, Seattle; 3 Immunotherapy Laboratory, Laboratory for Translational Immunology, University Medical Center Utrecht, The Netherlands; 4 Department of Pediatrics, University of Washington, Seattle Children’s Research Institute; 5 Department of Laboratory Medicine, University of Washington, Seattle; 6 Department of Biostatistics, University of Washington, Seattle; 7 Vaccine Research Center, National Institute of Allergy and Infectious Diseases, National Institutes of Health, Bethesda, Maryland; 8 Virology Division, Department of Infectious Diseases and Immunology, Faculty of Veterinary Medicine, Utrecht University, Utrecht, The Netherlands; 9 Nepal Nutrition Intervention Project–Sarlahi; 10 Department of International Health, Johns Hopkins Bloomberg School of Public Health, Baltimore, Maryland; 11 Cincinnati Children’s Hospital Medical Center, Ohio; 12 Department of Global Health, George Washington University, Washington, District of Columbia; 13 Laboratory of Translational Immunology, University Medical Center Utrecht, The Netherlands

**Keywords:** breast milk, maternal vaccination, IgG and IgA antibodies, respiratory syncytial virus, acute respiratory infection

## Abstract

**Background:**

Transplacental respiratory syncytial virus (RSV) antibody transfer has been characterized, but little is known about the protective effect of breast milk RSV-specific antibodies. Serum antibodies against the prefusion RSV fusion protein (pre-F) exhibit high neutralizing activity. We investigate protection of breast milk pre-F antibodies against RSV acute respiratory infection (ARI).

**Methods:**

Breast milk at 1, 3, and 6 months postpartum and midnasal swabs during infant illness episodes were collected in mother–infant pairs in Nepal. One hundred seventy-four infants with and without RSV ARI were matched 1:1 by risk factors for RSV ARI. Pre-F immunoglobulin A (IgA) and immunoglobulin G (IgG) antibody levels were measured in breast milk.

**Results:**

The median breast milk pre-F IgG antibody concentration before illness was lower in mothers of infants with RSV ARI (1.4 [interquartile range {IQR}, 1.1–1.6] log_10_ ng/mL) than without RSV ARI (1.5 [IQR, 1.3–1.8] log_10_ ng/mL) (*P* = .001). There was no difference in median maternal pre-F IgA antibody concentrations in cases vs controls (1.7 [IQR, 0.0–2.2] log_10_ ng/mL vs 1.7 [IQR, 1.2–2.2] log_10_ ng/mL, respectively; *P* = .58).

**Conclusions:**

Low breast milk pre-F IgG antibodies before RSV ARI support a potential role for pre-F IgG as a correlate of protection against RSV ARI. Induction of breast milk pre-F IgG may be a mechanism of protection for maternal RSV vaccines.

## Teaser

Low breast milk prefusion (pre-F) immunoglobulin G (IgG) antibodies before respiratory syncytial virus (RSV) acute respiratory infection (ARI) supports a potential role for pre-F IgG as a correlate of protection against RSV ARI. Induction of breast milk pre-F IgG may be a mechanism of protection for maternal RSV vaccines.

Maternal vaccination against respiratory syncytial virus (RSV) is a promising intervention to protect young infants against RSV infection through transfer of antibodies from mother to infant [1]. Transplacental transfer of RSV immunoglobulin G (IgG) antibodies via the neonatal Fc receptor has been characterized in mother–infant pairs in different populations [2–5]. Transplacental transfer ratio and decay kinetics of maternal IgG are considered cornerstones of protection of the infant through maternal vaccination [6]. However, other routes of antibody transfer may also be important to protect infants from RSV disease.

A novel route of RSV antibody transfer directly to the respiratory tract via RSV-specific IgG and immunoglobulin A (IgA) in amniotic fluid was recently described [7]. The acquired amniotic fluid antibodies show neutralizing activity against RSV and provide protection to the neonate for at least 1 week postpartum in vivo, demonstrating the role of mucosal immunity in protection of infants.

Postnatal antibody transfer to the mucosal surfaces occurs via breast milk [8–12]. A better understanding of the role of RSV-specific antibodies in breast milk may give further insight into mucosal antibody transfer from mother to infant in the context of maternal vaccination and may serve as a correlate of protection against RSV disease. Correlates of protection for RSV remain a knowledge gap and priority for RSV vaccine development [13]. Despite the lack of a clear correlate of protection [14], recent insights into the structure of viral envelope proteins have led to the distinction in antibody function on the basis of target epitopes. RSV F protein mediates RSV entry and fusion with the host cell membrane. Antibodies that target prefusion F (pre-F) protein account for the majority of neutralizing activity against RSV in human sera of infected individuals [15–17] and modify disease severity in young children [18]. Thus, antibodies directed against pre-F play an important role in protection against RSV infection. No previous studies have evaluated pre-F RSV antibody in breast milk in relationship to RSV disease risk in infants.

The aim of this study was to characterize the relationship between pre-F antibodies in breast milk and RSV acute respiratory infection (ARI) in infants.

## METHODS

### Study Site, Design, and Population

From mid-April 2011 to mid-April 2013, 3693 women in the second to third trimester of pregnancy were enrolled in a maternal influenza immunization trial in rural southern Nepal [19]. Weekly home-based visits were conducted until 180 days after birth for respiratory symptom surveillance of mother–infant pairs based on maternal report of symptoms each day in the past week. Nasal swabs were collected from infants if respiratory illness was noted; samples from mothers were collected for febrile respiratory disease. Breast milk was collected from a subset of 827 women living in the 3 study regions closest to the study clinic. Within this subset of mother–infant pairs, infants who had RSV-confirmed respiratory illness in the first 6 months of life were matched 1:1 to controls (infants with no RSV ARI) based on the following risk factors for RSV ARI: maternal influenza vaccination, maternal education, infant month of birth, number of siblings, use of an indoor biomass cook stove, and preterm birth (<37 weeks gestational age). Healthy infant controls were matched to have at least 4 months of respiratory surveillance.

### Data Collection and Case Definition

A respiratory illness was defined as fever, cough, wheezing, rapid breathing, or a draining ear on any 1 day in the past week. Breastfeeding was not exclusive if anything other than breast milk was given to the baby. Illness episodes were considered distinct when separated by 7 symptom-free days. Clinical and sociodemographic data were collected at enrollment, birth, and weekly respiratory surveillance visits. Midnasal swabs were collected from infants who met criteria for respiratory illness in the past 7 days and were tested for RSV by reverse-transcription quantitative polymerase chain reaction (PCR) [20]. Breast milk was collected at 1, 3, and 6 months postpartum. Participants were asked to wash their hands and self-express 15 mL of breast milk into a sterile container. Samples were transported on wet ice to the field laboratory and centrifuged to remove the lipid layer, aliquoted, and frozen at –80°C prior to shipment to the University of Washington (Seattle) for testing.

### Laboratory Testing

Breast milk IgA and IgG antibody concentrations against RSV-stabilized pre-F (DS-Cav1) protein were quantified by enzyme-linked immunosorbent assay (ELISA). DS-Cav1 is an RSV F protein that is stabilized by a T4 fibritin-trimerization domain (foldon) at the C-terminal, S155C, and S290C cysteine mutations to form an additional disulfide bond, and S190F and V207L cavity-filling mutations. DsCav-1 is expressed by transient transfection of HEK293F cells and purified by affinity purification (NTA resin and StrepTactin resin) and a Superose 200 gel filtration column [21]. Nunc MaxiSorp 96-well plates (Thermo Scientific) were coated overnight at 4°C with either pre-F (100 ng/mL, DS-Cav1, for pre-F IgA or pre-F IgG ELISA). In between steps, plates were washed 3 times with phosphate-buffered saline (PBS) containing 0.05% Tween-20 (Sigma-Aldrich) (PBS-T) using a microplate washer (Biotek 405 LS). Plates were blocked for 1 hour at room temperature with 1% bovine serum albumin (Roche Diagnostics) in PBS-T. Breast milk was added (100 μL/well) in duplicate, at 2–3 dilutions and incubated for 1.5 hours at room temperature. Recombinant palivizumab IgA1 and recombinant palivizumab IgA2 were synthesized by cloning variable heavy and light chain sequences of palivizumab into Lonza expression vector, followed by production in HEK293T cells, and purification by KappaSelect and high pressure size exclusion chromatography [22]. Recombinant palivizumab IgA1 and IgA2 and palivizumab (Synagis, MedImmune) were used to generate a standard curve on every plate. Horseradish peroxidase–labeled goat antihuman IgA and horseradish peroxidase–labeled goat-anti-human IgG (both Jackson ImmunoResearch) were added at a concentration of 0.5 μg/mL and 0.16 μg/mL, respectively, as detection antibodies and incubated for 1 hour at room temperature. Plates were developed with ABTS substrate (Roche) and absorbance was measured at 415 nm with a microplate spectrophotometer (Biotek Epoch). Data were captured and exported using Gen5 software (Biotek).

### Statistical Analyses

Continuous variables were described using mean (standard deviation [SD]) or median (interquartile range [IQR]). Differences in the mean or median of continuous variables were tested with a 2-sided *t* test or a nonparametric Mann–Whitney test when appropriate. Log_10_ transformation was performed for all antibody measurements. For our primary analysis, we compared antibody titers prior to infection using a Mann–Whitney test; for infections that occurred before 1 month, we used the antibody titer at 1 month. We used the corresponding time point for controls as used for the matched cases. A linear mixed-model analysis was performed to compare the difference of antibody titers over time for cases and controls. We included time of breast milk collection (month 3 or 6 vs month 1) as covariates and RSV status of children in the first 6 months of life, as well as the interaction terms of collection time by RSV positivity, to test the hypothesis of whether RSV antibody levels in breast milk increased or decreased differently by RSV status of the infant. We used a Spearman correlation to perform a correlation of RSV antibody titer to time of infection in cases only, as well as a correlation of pre-F antibody to total antibody by isotype and pre-F IgA to pre-F IgG in both cases and controls. The statistical analysis was performed using Stata/SE 13.1 software (StataCorp) and sinusoid function to examine seasonal variation using SPSS Statistics 25 (IBM) software.

### Ethical Considerations

Ethical approval for the primary trial (ClinicalTrials.gov identifier NCT01034254) was obtained from the institutional review boards at the Institute of Medicine at Tribhuvan University, the Nepal Health Research Council, John Hopkins University Bloomberg School of Public Health, Seattle Children’s Hospital, and Cincinnati Children’s Medical Center.

## RESULTS

### Clinical and Sociodemographic Characteristics

Clinical and sociodemographic characteristics were compared for 174 children (87 cases and 87 controls). One hundred six of the 174 children (61%) were female. No significant differences for clinical or sociodemographic characteristics of mothers or infants were observed between cases and controls (Table 1). The mean age at primary RSV ARI in cases was 3.1 (SD, 1.5) months.

**Table 1.** Maternal and Pediatric Clinical Characteristics of Cases and Controls

Characteristic	Cases (n = 87)	Controls (n = 87)	*P* Value
Maternal			
Median age, y (IQR)	22 (19–27)	22 (20–26)	.64
Mean body mass index, kg/ m^2^ (SD)	21.0 (2.5)	20.7 (2.9)	.55
Literacy	47/82 (57.3)	47/81 (58.0)	.93
Nulliparous	31/87 (35.6)	35/87 (40.2)	.53
Exclusive breastfeeding	57/86 (66.3)	49/87 (56.3)	.21
Household smoking	3/82 (3.7)	4/81 (4.9)	.72
Influenza vaccination^a^	40/87 (46.0)	40/87 (46.0)	.99
No. of respiratory episodes during pregnancy	5/87 (5.8)	6/87 (6.9)	.76
No. of respiratory episodes after delivery	6/87 (6.9)	4/87 (4.6)	.52
Pediatric			
Mean age at RSV illness, mo (SD)	3.1 (1.5)	NA	NA
Mean birth weight, g (SD)	2767 (401)	2802 (488)	.63
Low birth weight	15/75 (20.0)	20/74 (27.0)	.31
Median gestational age, wk (IQR)	40 (39–41)	40 (39–41)	.33
Small for gestational age	35/75 (46.7)	30/74 (40.5)	.45
Preterm^a^	6/87 (6.9)	6/87 (6.9)	.99
Female sex	40/87 (46.0)	38/87 (43.7)	.76

Data are presented as no./No. (%) unless otherwise indicated. Baseline characteristics of children with and without RSV acute respiratory infection in the first 6 months of life (cases and controls) are shown. Maternal and pediatric clinical and sociodemographic characteristics were compared between cases and controls. The Intergrowth-21 criteria [46] were used to calculate small for gestational age. Differences in mean/median of continuous variables were tested with the 2-sided *t* test or a nonparametric Mann–Whitney test when appropriate. Categorical variables were described with frequencies and percentages and compared between groups using χ^2^ test.

Abbreviations: IQR, interquartile range; NA, not applicable; RSV, respiratory syncytial virus; SD, standard deviation.

^a^Variables used to match controls to cases.

### Quantification of Pre-F IgA, Pre-F IgG, Total IgA, and Total IgG

Pre-F IgA, pre-F IgG, total IgA, and total IgG antibodies were measured in 454 breast milk samples from 174 mothers at 1 month (n = 150), 3 months (n = 151), and 6 months (n = 153) postpartum. The median concentration of pre-F IgA (77.7 [IQR, 22.3–200.7] ng/mL) was higher than the median concentration of pre-F IgG (36.5 [IQR, 21.0–62.8] ng/mL). Likewise, the median concentration of total IgA was higher (0.2 [IQR, 0.15–0.27] mg/mL) than total IgG (0.04 [IQR, 0.03–0.05] mg/mL) (Supplementary Table 1). In Table 2 the log_10_ median concentrations of pre-F IgA, pre-F IgG, total IgA, and total IgG for all breast milk samples for both cases and controls are described in addition to the raw values in Supplementary Table 1.

**Table 2.** Antibody Measured in Breast Milk at All Time Points Combined, Log-Adjusted Data

Breast Milk Antibody Measured	All (N = 454), Log_10_ ng/mL		Cases (n = 227), Log_10_ ng/mL		Controls (n = 227), Log_10_ ng/mL	
	Median	(IQR)	Median	(IQR)	Median	(IQR)
Pre-F IgA (n = 450)	1.9	(1.3–2.3)	2.0	(1.4–2.3)	1.8	(1.3–2.2)
Pre-F IgG (n = 449)	1.6	(1.3– 1.8)	1.5	(1.3– 1.7)	1.6	(1.4–1.8)
Total IgA (n = 452)	5.3	(5.2–5.4)	5.3	(5.2–5.4)	5.3	(5.2–5.4)
Total IgG (n = 447)	4.5	(4.4–4.7)	4.5	(4.4–4.7)	4.6	(4.4–4.7)

Table shows median antibody titers in breast milk of all 174 mothers, and cases and controls separately for all time points combined. Log_10_ pre-F IgA, pre-F IgG, total IgA, and total IgG concentrations are shown for all children, and cases and controls separately. Pre-F IgA and pre-F IgG antibodies are expressed as nanograms per milliliter. Total IgA and total IgG antibodies are measured in milligrams per milliliter.

Abbreviations: IgA, immunoglobulin A; IgG, immunoglobulin G; IQR, interquartile range; Pre-F, prefusion F.

### Correlation Between Specific and Total Antibody Levels

Pre-F IgG concentration showed a moderate positive correlation to total IgG at 1 month (ρ = 0.38; *P* < .0001; Supplementary Figure 2), 3 months (ρ = 0.38; *P* < .0001), and 6 months (ρ = 0.40; *P* < .0001) postpartum. Pre-F IgA showed a lower positive correlation to total IgA at 1 month (ρ = 0.22; *P* = .007; Supplementary Figure 2) and at 3 months (ρ = 0.27; *P* = .0009), but not at 6 months (ρ = 0.09; *P* = .29). Pre-F IgG was positively correlated with pre-F IgA at 1 month (ρ = 0.18; *P* = .03; Supplementary Figure 3), at 3 months (ρ = 0.37; *P* < .0001), and at 6 months (ρ = 0.22; *P* = .008) postpartum.

### Pre-F IgG and Pre-F IgA in Cases and Controls Before Infection

**Figure 1.**
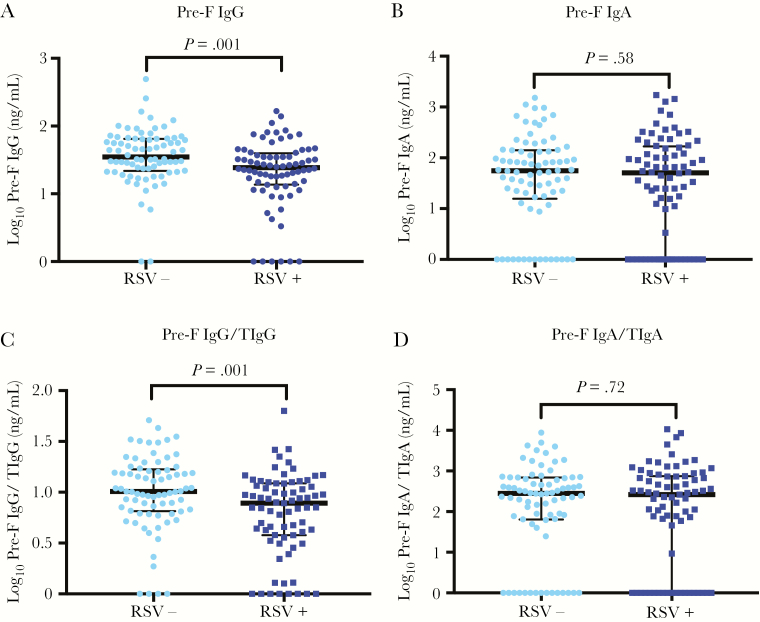
Prefusion F (pre-F) antibody titers prior to time of infection in cases (respiratory syncytial virus positive [RSV+]) and matched controls (respiratory syncytial virus negative [RSV–]). Mann–Whitney test was performed to compare medians of cases and controls. Pre-F antibody titer was compared for measurement prior to infection. For healthy controls, antibody measurement at time of infection for age-matched cases was used. Ratio of pre-F immunoglobulin A (IgA) to total IgA (TIgA) was multiplied by 1 × 10^6^ to ensure values on the y-axis were >0. Ratio of pre-F immunoglobulin G (IgG) and total IgG (TIgG) was multiplied by 1 × 10^4^ for the same reason. *A*, Log_10_ pre-F IgG. *B*, Log_10_ pre-F IgA. *C*, Log_10_ pre-F IgG divided by log_10_ TIgG. *D*, Log_10_ pre-F IgA divided by log_10_ TIgA.

We compared pre-F antibody titers at the time point prior to RSV ARI in cases and matched controls. If there was no breast milk sample before infection, then we used the closest time point after RSV ARI. The median time gap between antibody measurement used and RSV ARI was 1.1 (IQR, 0.45–1.6) months. Eight infants had RSV ARI before 1 month of age, and the median time at RSV ARI in these infants was age 0.64 (IQR, 0.49–0.80) months. The median log_10_ pre-F IgG antibody titer before infection was significantly lower in breast milk of mothers of cases (median, 1.4 [IQR, 1.1–1.6] log_10_ ng/mL) than in mothers of controls (median, 1.5 [IQR, 1.3–1.8] log_10_ ng/mL) (*P* = .001; Figure 1A). The effect was more pronounced after excluding 8 children who had RSV ARI before 1 month of age and their matched controls: The log_10_ pre-F IgG antibody titer was significantly lower in breast milk of mothers of cases (median, 1.4 [IQR, 1.1–1.5] log_10_ ng/mL) compared with mothers of controls (median, 1.6 [IQR, 1.3–1.8] log_10_ ng/mL) (*P* = .0002; Supplementary Figure 5*A*).

The median log_10_ pre-F IgA antibody titer of the breast milk sample at the latest time point prior to infection did not differ significantly in breast milk of mothers of cases (median, 1.7 [IQR, 0.0–2.2] log_10_ ng/mL) compared with mothers of controls (median, 1.7 [IQR, 1.2–2.1] log_10_ ng/mL) (*P* = .58; Figure 1B). Similarly, when excluding children with RSV ARI <1 month of age, there was no significant difference in pre-F IgA antibody in breast milk of mothers of cases (median, 1.7 [IQR, 0.0–2.1] log_10_ ng/mL) compared with mothers of controls (median, 1.7 log_10_ ng/mL [IQR, 1.1–2.1] log_10_ ng/mL) (*P* = .50; Supplementary Figure 5*B*).

We evaluated the ratio of pre-F antibody titers to total antibody titer by IgG or IgA isotype. For the ratio of pre-F IgG to total IgG and pre-F IgA levels to total IgA, the same trends were observed (Figure 1C and 1D). Pre-F IgG/total IgG was lower in cases than in controls (0.89 [IQR, 0.58–1.1] log_10_ ng/mL vs 1.0 [0.83–1.2] log_10_ ng/mL; *P* = .001), whereas pre-F IgA/total IgA did not differ between cases and controls (2.4 [0.0–2.9] log_10_ ng/mL vs 2.4 [1.8–2.8] log_10_ ng/mL; *P* = .72). We performed a sensitivity analysis for infants who were exclusively breastfed in the first few days of life (n = 106), and found that pre-F IgA prior to infection did not differ significantly between cases and controls (1.7 [1.1–2.2] log_10_ ng/mL vs 1.9 [0.1–2.1] log_10_ ng/mL; *P* = .85), but did differ for pre-F IgG (1.3 [1.1–1.6] log_10_ ng/mL vs 1.6 [1.3–1.8] log_10_ ng/mL; *P* = .01).

### Mixed-Model Analysis of Pre-F Antibodies Over Time

**Figure 2.**
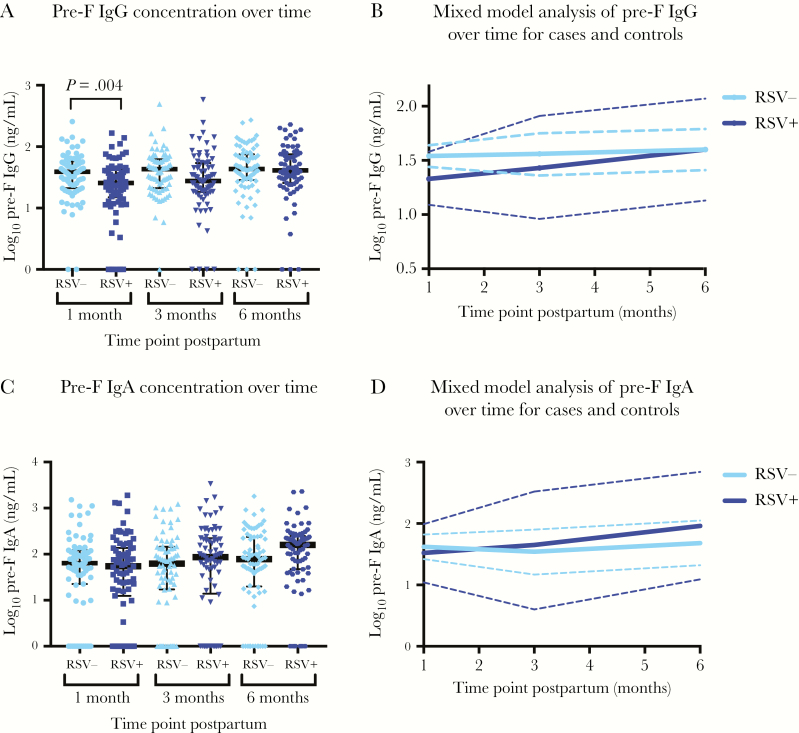
Mixed-model analysis of prefusion F (pre-F) antibody in cases and controls over time. A linear mixed-model analysis was used to examine the effect of respiratory syncytial virus (RSV) on pre-F antibodies at different time points. *A*, Log_10_ pre-F immunoglobulin G (IgG) antibody concentration at 1, 3, and 6 months postpartum for cases (RSV positive [RSV+], dark blue) and controls (RSV negative [RSV–], light blue), with medians indicated in black. *B*, Linear mixed-model analysis for log_10_ pre-F IgG in cases and controls. Solid line is the mean, and dashed line indicates the 95% confidence interval (CI). *C*, Log_10_ pre-F immunoglobulin A (IgA) antibody concentration at 1, 3, and 6 months postpartum for cases and controls, with medians in black. *D*, Linear mixed-model analysis for log_10_ pre-F IgA in cases and controls. Solid line is the mean, and dashed line indicates the 95% CI.

We used a mixed-effects linear regression model to compare pre-F IgG and pre-F IgA antibody concentrations in breast milk over time in mothers of cases compared to controls. The mean log_10_ difference of pre-F IgG concentration in breast milk of mothers of cases compared to controls was –0.21 (95% confidence interval [CI], –.35 to –0.06; *P* = .004) at 1 month postpartum, –0.12 (95% CI, –.26 to .02; *P* = .09) at 3 months postpartum, and 0.00 (95% CI, –.14 to .14; *P* = .99) at 6 months postpartum (Figure 2A and 2B)_._ The mean log_10_ difference of pre-F IgA in breast milk of mothers of cases compared to controls was –0.10 (95% CI, –.38 to .17; *P* = .46) at 1 month postpartum, 0.11 (95% CI, –.17 to .38; *P* = .44) at 3 months postpartum, and 0.28 (95% CI, .01–.55; *P* = .046) at 6 months postpartum (Figure 2C and 2D). Antibody level was found to increase at 6 months relative to month 1 only for cases (0.27 log_10_ ng/mL increase in titer for pre-F IgG, *P* = .001; 0.44 log_10_ ng/mL increase for pre-F IgA, *P* = .003). There was no evidence of increase over time for either antibody level among controls (*P* = .45 and *P* = .21 for pre-F IgA and pre-F IgG, respectively). Consequently, for pre-F IgG, the difference found at 1 month between cases and controls was no longer present at 6 months of age (*P* = .99).

### Antibody Concentration and Time to Infection

Among cases, there was a low negative correlation between pre-F IgG concentration in breast milk at 1 month postpartum and time to RSV ARI in cases, which is marginally significant (ρ = –0.22; *P* = .06), indicating that higher antibody levels may be associated with shorter time to infection. However, there was no detected correlation between pre-F IgA antibody concentration at 1 month postpartum and time to RSV ARI in cases (ρ = 0.10; *P* = .40).

### Seasonal Fluctuation of Pre-F IgA and Pre-F IgG Titers

We applied a sinusoidal model to the pre-F IgG and pre-F IgA concentrations in breast milk of all mothers at 1 month postpartum. All RSV-infected infants in this substudy were born between June and September, as were the controls who were matched by birth month. Therefore, no children in this substudy were born in October through January, corresponding to the peak of the RSV season in Nepal [23], which resulted in a poor fit of the model (goodness-of-fit measure: *r* = 0.008 for pre-F IgG; *r* = 0.02 for pre-F IgA) (Supplementary Figure 4*A* and 4*B*).

## DISCUSSION

We provide evidence that IgG antibodies in breast milk against RSV pre-F are lower in mothers of children who develop RSV ARI in the first months of life compared with children who do not. In the context of RSV maternal vaccine development with no established correlate of protection against RSV [24], we conclude that breast milk pre-F IgG antibodies may be a correlate of protection against RSV ARI. The importance of these findings is underscored by the fact that premature infants, who are disproportionately affected by RSV disease [25] and have reduced transplacental antibody transfer, may still be potentially protected by maternal immunization via breast milk [11].

One strength of this study was the development of a novel breast milk RSV antibody assay targeting the RSV fusion protein in a pre-F–stabilized conformation, which permitted measurement of antibodies known to be an important target for RSV neutralizing antibodies [15]. Additionally, the use of recombinant palivizumab IgA allowed for accurate measurement with an IgA standard. Palivizumab IgA1 and IgA2 were used in a 3:2 ratio as found in human breast milk [26].

The results show a potential protective role against RSV ARI for breast milk pre-F IgG but not pre-F IgA antibodies. The difference in pre-F IgG between cases and controls is small, though statistically significant. The difference in protection across antibody isotypes is in accordance with studies specific to RSV and other pathogens, such as human immunodeficiency virus [27] and cytomegalovirus [28]. Likewise, recombinant palivizumab IgA offers less effective protection following intranasal administration than IgG in mice [22].

The relationship between breast milk pre-F IgG and time to infection was an exploratory analysis; the negative correlation merits further study in a larger population powered to look at this effect using more frequent sequential breast milk samples collected over time and a comparison to serologic assays. When looking at seasonality of breast milk pre-F antibodies in breast milk, IgA but not IgG decreased in the summer months, possibly due to the shorter half-life [29] and lack of exposure to RSV. Increases in breast milk pre-F IgG at 6 months postpartum may have reflected exposure and infection of the mothers. However, in our study we did not sample for respiratory viruses in asymptomatic or afebrile illnesses in women, therefore limiting our ability to detect these by molecular diagnosis.

There is consensus on the protective effect of breastfeeding on infant respiratory morbidity and mortality [30], with lower risk of RSV hospitalization and reduced disease severity when comparing breastfed to nonbreastfed infants [31–33]. However, evidence for the mechanisms by which breast milk antibodies may enter the neonatal circulation is limited. Breast milk antibodies have been shown to reach the neonatal circulation in 3 children who were given antibody-rich human colostrum via a nasogastric tube [34]. After closure of the gut, uptake of IgG may occur via the neonatal Fc receptor, which has been identified in the human intestine [35] and is involved in bidirectional transport across the enterocyte, allowing for defense at the mucosal level [36]. Secretory IgA plays a role at the mucosal surface by neutralizing pathogens in the intestinal lumen in humans [36].

Boosting breast milk antibody via maternal vaccination may help protect infants from RSV disease. In a subunit RSV vaccine trial, increased IgA and IgG antibodies against RSV in breast milk were measured in vaccinated compared to nonvaccinated women [4]. Increased concentrations of breast milk antigen-specific antibodies have been measured following maternal vaccination against influenza, pertussis, RSV, and *Streptococcus pneumoniae* [37]. Only 1 study examined the association between respiratory pathogen–specific antibodies and clinical outcomes in 57 infants [38]. In this study, maternal influenza vaccination and increased influenza-specific IgA in breast milk correlated with decreased episodes of infant respiratory illness, though IgG was not measured. Finally, there is evidence that high virus-specific IgA may interfere with vaccine response for rotavirus vaccination [39], which may be a consideration for RSV maternal vaccination.

The most important limitation of this study was that we did not measure pre-F antibodies in serum of all mothers of these infants or in cord blood. No blood was drawn from infants during this study, so further study in infants was not possible. An alternative explanation for protection may be serum pre-F antibody titers in women and their infants. However, in a subset of 310 maternal infant pairs within the maternal vaccination cohort, neutralizing RSV antibody titers in cord blood were not shown to protect against RSV ARI [40]. For 44 maternal infant pairs that overlapped with the cohort in this study, we examined the correlation between breast milk pre-F IgG at 1 month postpartum and cord blood antibody titers and found a positive correlation between breast milk pre-F IgG antibodies and neutralizing antibody titers in cord blood (*r*(^2^) = 0.29; *P* = .05). We found no relationship between breast milk pre-F IgA and cord blood neutralizing antibody titers (*r*(^2^) = –0.07; *P* = .6). In an exploratory analysis, we found no relationship between disease severity and breast milk pre-F IgG and IgA antibody titers from samples collected closest to the time of infection (data not shown). Furthermore, we found no relationship between breast milk pre-F antibody levels at the time points closest to infection and nasal swab PCR cycle threshold values (data not shown). Another limitation of this study is a facet of the study design. Although RSV ARI often occurs after 6 months of age [25, 41], in this study we were limited to early RSV infection in subjects <6 months of age by design. We did not measure antibody titers in the first month postpartum. The study population was small (N = 174), though larger than almost all studies measuring antigen-specific antibodies against respiratory pathogens (n = 5–258) [37] and larger than any study measuring RSV antibodies in breast milk (n = 57–130) [4, 42, 43]. These results should be replicated in a larger group in a different population with longer follow-up time. An additional limitation was the choice to measure antibodies against pre-F but not to exclude antibodies that bind epitopes present on postfusion F protein (sites II and IV). Antibodies that target only antigenic site ø show high neutralizing activity [44] and may correlate even better with protection from RSV. The protective effect of breast milk pre-F–specific antibodies against respiratory disease may have been underestimated because antibodies against all pre-F epitopes were measured, which included less potent RSV-neutralizing or nonneutralizing antibodies. Finally, we did not measure antibodies against G protein, which have recently also been shown to display neutralizing activity and correlate with decreased disease severity in infants and young children [45] and should be assessed in future studies.

In conclusion, the current study provides evidence that pre-F IgG antibodies in breast milk may play a protective role against RSV-confirmed ARI in the first 6 months of life. Induction of pre-F IgG in breast milk may be a potential mechanism of protection of maternal RSV vaccine candidates.

## Supplementary Data

Supplementary materials are available at *The Journal of Infectious Diseases* online (http://jid.oxfordjournals.org/). Supplementary materials consist of data provided by the author that are published to benefit the reader. The posted materials are not copyedited. The contents of all supplementary data are the sole responsibility of the authors. Questions or messages regarding errors should be addressed to the author.

Supplemental Materials

## Notes

***Author contributions.*** All co-authors contributed to the final manuscript. M. C. S., J. M. T., J. K., S. K. K., and J. A. E. contributed to the design of the original trial. M. C. S. conceived of and secured funding for the original trial. S. K., S. L., J. M. T., and J. K. supervised the conduct of the study in the field for the original trial.

***Acknowledgments.*** We thank Marijn Hazelbag and Christiana Naaktgeboren for assistance with the power analysis; Marijn Hazelbag and Julia Drylewicz for critical review of statistical analysis and consulting on statistical methods; Shari Cho for aiding with stability testing of antibodies in breast milk; and Margaret Green for provision of the de-identified samples for assay development.

***Financial support.*** This work was supported by the Netherlands Royal Academy for Medical Sciences (Academy medical sciences grant 2017, Academy Medical Sciences Fund); the Bill & Melinda Gates Foundation (grant number 50274); and the National Institutes of Health (grant number K23-AI103105).

***Potential conflicts of interest.*** H. Y. C.’s institution receives research support from Novavax and Sanofi-Pasteur. J. A. E. has received research support to her institution from Novavax, MedImmune, GlaxoSmithKline, and Gilead. B. S. G. has a patent pending for an RSV pre-F candidate. J. M. T. and J. K. received grants from the Bill & Melinda Gates Foundation (BMGF) during the conduct of the study. J. K. has received grants from BMGF, the Thrasher Research Fund, the National Institutes of Health, the Whole New World Foundation, and the US Agency for International Development. L. J. B. has regular interaction with pharmaceutical and other industrial partners, but has not received personal fees or other personal benefits; and has received minor funding for consultation and invited lectures by AbbVie, MedImmune, Ablynx, Bavaria Nordic, MabXience, Novavax, and Janssen (total annual estimate <€20000). University Medical Center Utrecht has received major funding (>€100000 per industrial partner) for investigator-initiated studies from AbbVie, MedImmune, Janssen, BMGF, and MeMed Diagnostics, as well as minor funding for participation in trials by Regeneron and Janssen since 2015 (total annual estimate <€20000). All other authors report no potential conflicts of interest. All authors have submitted the ICMJE Form for Disclosure of Potential Conflicts of Interest. Conflicts that the editors consider relevant to the content of the manuscript have been disclosed.

## Notes

Presented in part: RSV Vaccines for the World Meeting, 29 November–1 December 2017, Malaga, Spain.
