# Supplementary material for: Breast Milk Prefusion F Immunoglobulin G as a Correlate of Protection Against Respiratory Syncytial Virus Acute Respiratory Illness
Source: J Infect Dis. 2018 Aug 10;219(1):59–67. doi: 10.1093/infdis/jiy477 (PMC6284547; doi:10.1093/infdis/jiy477)
Supplement: Supplemental Materials [file jiy477_suppl_supplemental_materials.docx]

Supplementary Appendix Table/Figure Legends

**Table S1.** Antibody titers in breast milk of 174 mothers and cases and controls separately for all 3 time points combined shown as raw values. Pre-F IgA, pre-F IgG, total IgA, and total IgG concentrations for all mothers, and cases and controls separately. Pre-F IgA and pre-F IgG are measured in nanograms/milliliter (ng/mL) and total IgA and total IgG in milligrams/milliliter (mg/mL).

**Figure S1.** Correlation of pre-F antibodies in breast milk at 1 month postpartum with time to RSV ARI.

Spearman’s correlation with r(s)= Spearman’s correlation coefficient. (A) Log_10_ pre-F IgG in breast milk at 1 month postpartum was correlated to time to RSV ARI in months (B) Log_10_ pre-F IgA in breast milk at 1 month postpartum was correlated to time to RSV ARI in months.

**Figure S2.** Correlation of pre-F antibodies to total antibodies by isotype in breast milk at 1 month postpartum.

Spearman’s correlation with r(s)= Spearman’s correlation coefficient. (A) Correlation of log_10_ pre-F IgA at 1 month postpartum to log_10_ total IgA at 1 month postpartum (B) Correlation of log_10_ pre-F IgG at 1 month postpartum and log_10_ total IgG at 1 month postpartum.

**Figure S3.** Correlation of pre-F IgA with pre-F IgG antibodies in breast milk. Spearman’s correlation with r(s)= spearman’s correlation coefficient. Log_10_ pre-F IgA at 1 month postpartum was correlated to Log_10_ pre-F IgG at 1 month postpartum in 174 mothers.

**Figure S4.** Concentration of pre-F antibodies in breast milk in relation to birth month.

We fit a sinusoidal model to antibody concentrations by birth month. Predicted antibody levels are shown with the solid line. True antibody levels shown by bar graph. (A) Log_10_ pre-F IgG true and predicted antibody levels by birth month (B) Log_10_ pre-F IgA true and predicted antibody levels by birth month.

Figure S5. Pre-F antibody titers prior to time of infection in cases (RSV +) and matched controls (RSV –) excluding RSV infections before 1 month postpartum.

Mann-Whitney test was performed to compare medians of cases and controls. Pre-F antibody titer was compared for measurement prior to infection excluding infections occurring before 1 month postpartum (8 infants). For healthy controls, antibody measurement at time of infection for age matched case was used (excluding 8 matched controls). Ratio of pre-F IgA to total IgA was multiplied by 1x10^6^ to ensure values on the y-axis were greater than 0. Ratio of pre-F IgG and total IgG was multiplied by 1x10^4^ for the same reason. (A) Log_10_ pre-F IgG (B) Log_10_ pre-F IgA (C) Log_10_ pre-F IgG divided by log_10_ total IgG (D) Log_10_ pre-F IgA divided by log_10_ total IgA.

Supplementary Appendix

**Table S1. Antibody measured in breast milk at all time points combined, raw data**

|  | All (n=454) | | Cases (n=227) | | Controls (n=227) | |
| --- | --- | --- | --- | --- | --- | --- |
| Breast milk antibody measured | Median | IQR | Median | IQR | Median | IQR |
| Prefusion IgA  ng/mL  (n=450) | 77.7 | 22.3 – 200.7 | 90.3 | 24.6 – 216.9 | 65.9 | 20.7 – 143.0 |
| Prefusion IgG  ng/mL  (n=449) | 36.5 | 21.0 – 62.8 | 31.8 | 19.2 – 55.6 | 42.9 | 23.7 – 64.9 |
| Total IgA  mg/mL  (n=452) | 0.20 | 0.15 – 0.27 | 0.19 | 0.14 – 0.26 | 0.20 | 0.15 – 0.28 |
| Total IgG  mg/mL  (n=447) | 0.03 | 0.03 – 0.05 | 0.03 | 0.03 – 0.05 | 0.04 | 0.03 –0.05 |

Supplementary Appendix

**FIG S1.**


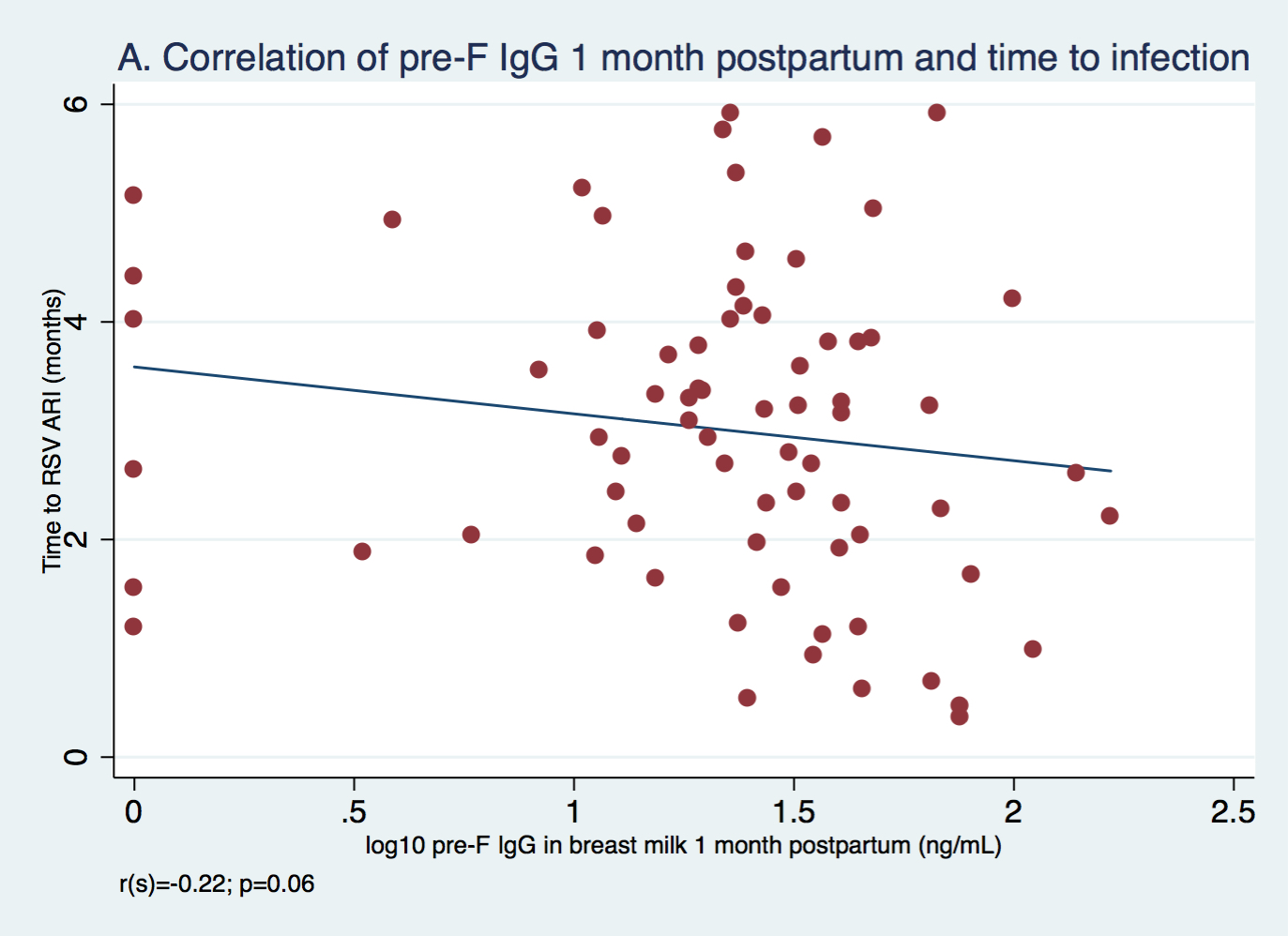

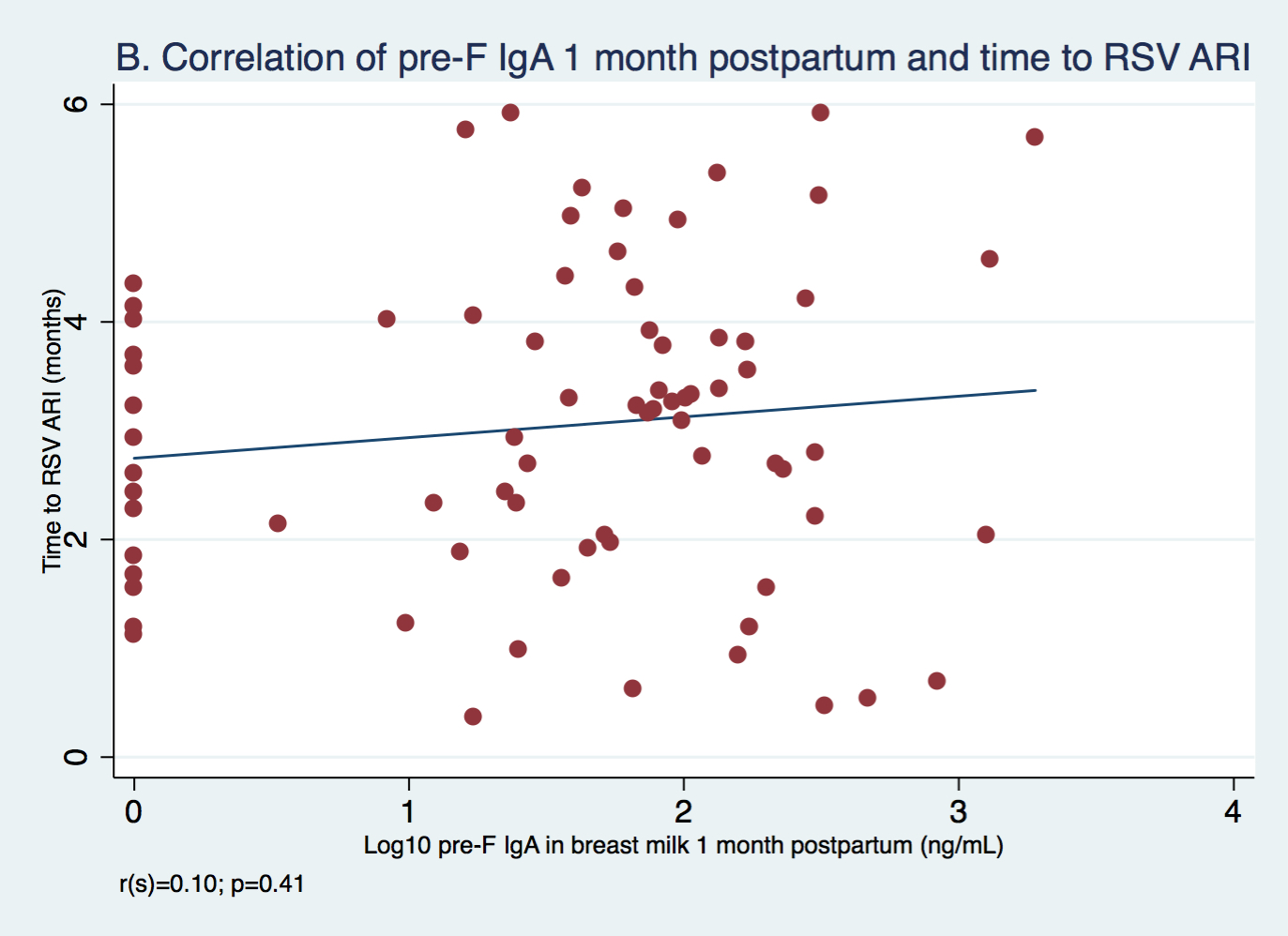


**FIG S2.**


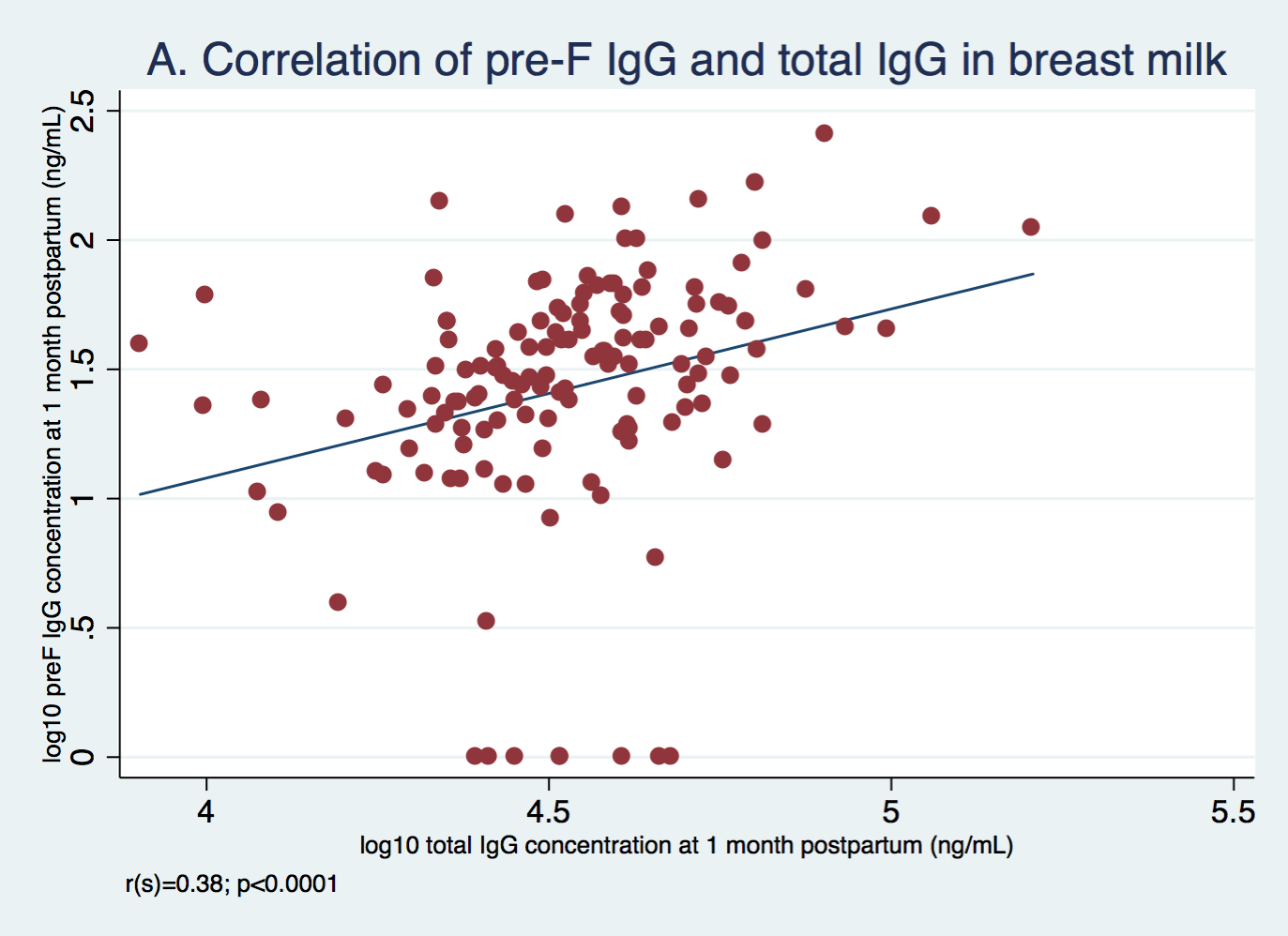

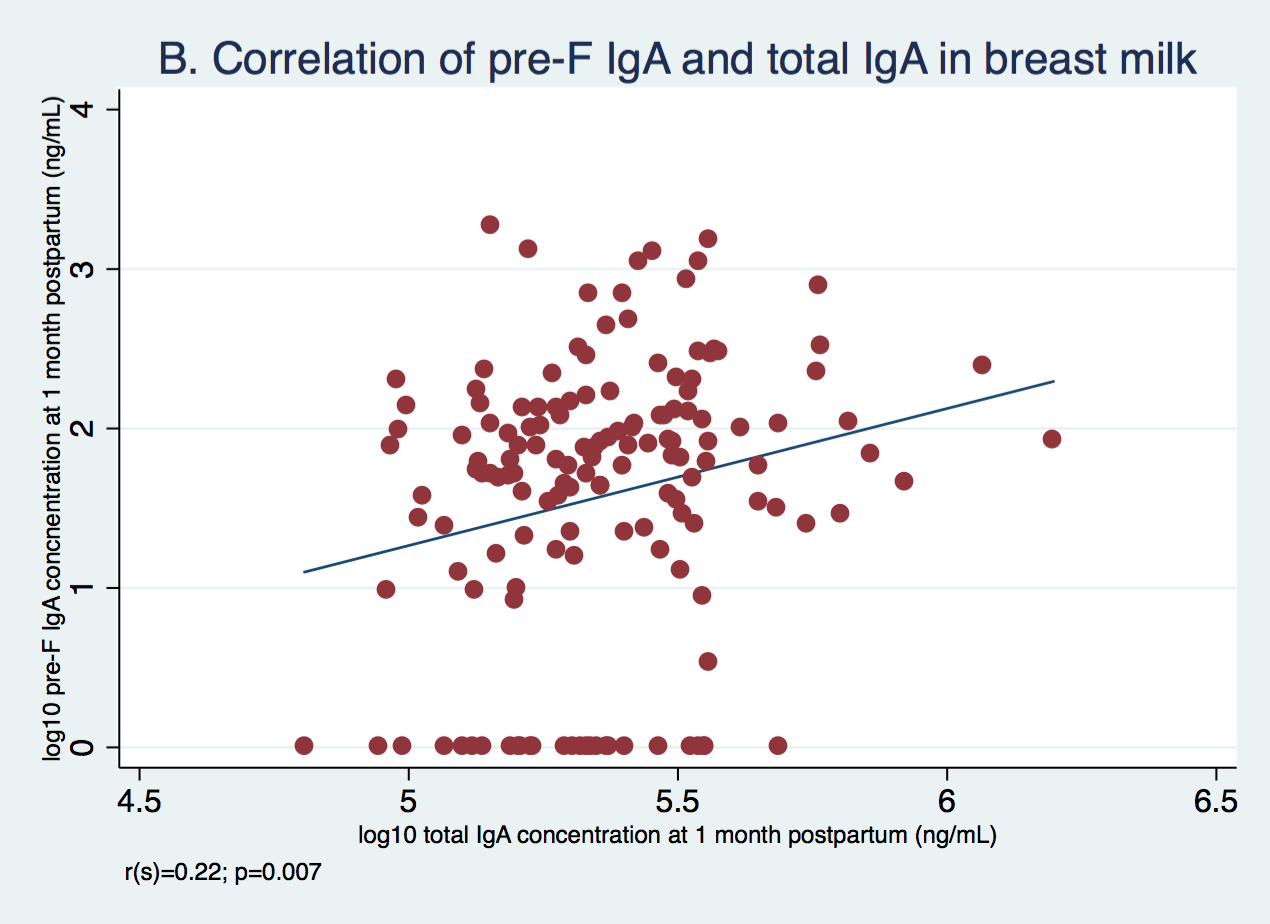


**FIG S3**

**
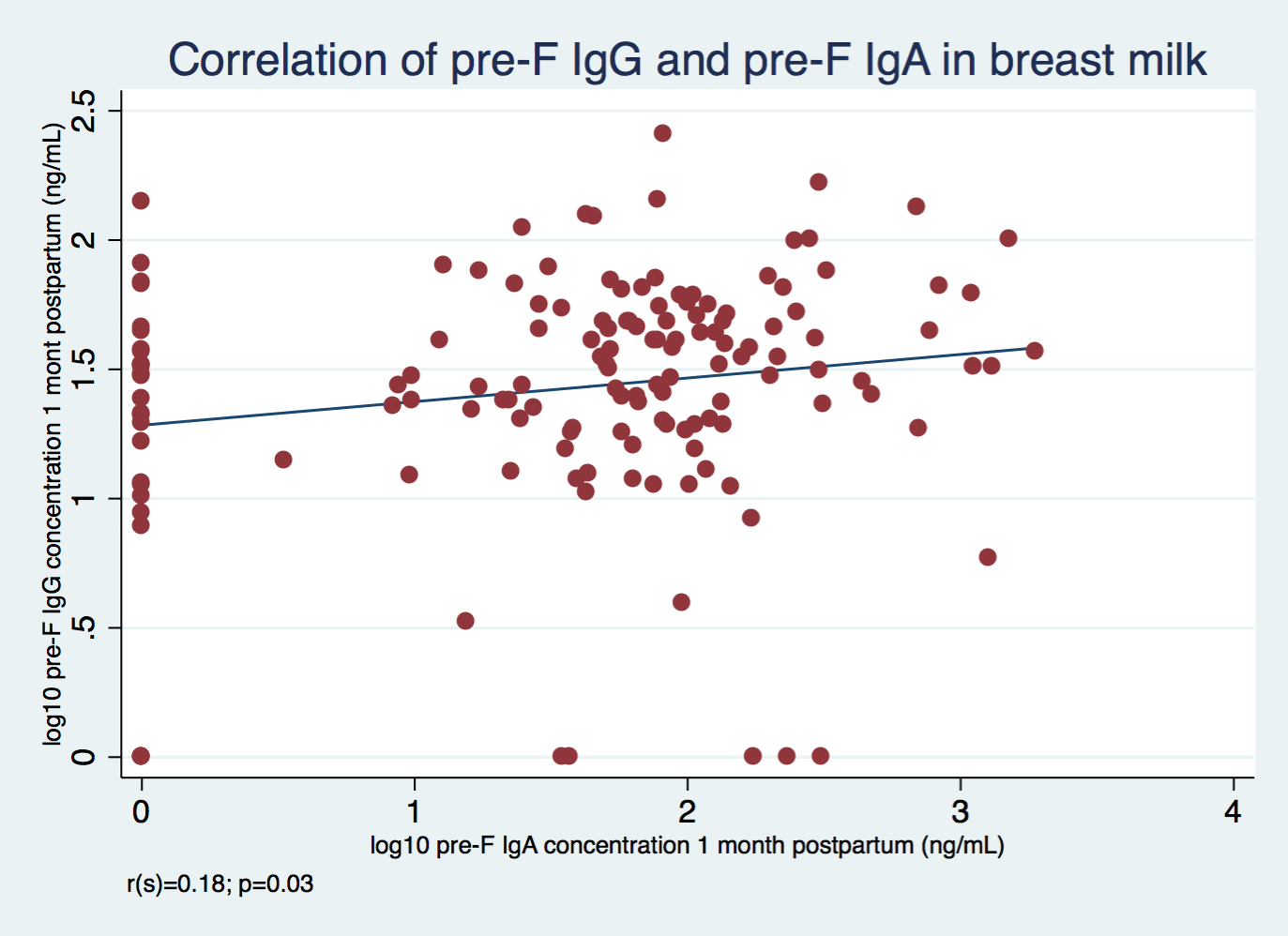
**

**FIG S4.**

**S5.**

*Supplemental Methods*

*Laboratory Testing*

Nunc MaxiSorp 96-well plates (Thermo Scientific, Roskilde, Denmark) were coated overnight at 4°C with 1:1 of goat anti-human kappa: goat-anti-human lambda antibodies (500 ng/mL Southern Biotech) for total IgA ELISA), or purified monoclonal human anti-IgG (100 μg/well, Thermofischer Scientific) for total IgG ELISA). In between steps plates were washed 3 times, or twice for total IgG ELISA, with PBS containing 0.05% Tween-20 (Sigma Aldrich) (PBS-T) using a microplate washer (Biotek 405 LS). Plates were blocked for 1 hour at room temperature (RT) with 1% bovine serum albumin (Roche Diagnostics) in PBS-T. Breast milk was added (100μl/well) in duplicate, at 2 to 3 dilutions and incubated for 1.5 hours at RT. Purified human IgA (Bethyl Laboratories Inc.; Montgomery, TX), and recombinant human IgG (Jackson, West Grove, PA) were used to generate a standard curve on every plate. Horseradish peroxidase-labeled goat-anti-human IgA (Jackson, West Grove, PA) and horseradish-peroxidase-labeled goat-anti-human IgG (Jackson, West Grove, PA) were added at a concentration of 0.5 μg/mL and 0.16 μg/mL, respectively, as detection antibodies and incubated 1 hour at RT. Plates were developed with ABTS substrate (Roche, Darmstadt, Germany) and absorbance was measured at 415nm with a microplate spectrophotometer (Biotek Epoch, Winooski VT), with the exception of total IgG which was developed with TMB solution (Thermofisher Scientific), stopped with hydrogen peroxide, and measured at 450nm. Data were captured and exported using Gen5 software (Biotek, Winooski VT).

*Quality Controls*

Only duplicate measurements with a coefficient of variation (CV) values under 20% were included in the final dataset for analysis. A 4-parameter logistic curve fit was used for the standard on each plate with a cut-off of R^2^>0.99 for goodness-of-fit. A pooled control breast milk sample was run on every plate; results were normalized to this standard to adjust for intra-assay variation. All samples were corrected for background absorbance. The linearity of dilution of each sample was considered acceptable if results were between 70-130% of the expected concentration, based on the concentration calculated from the previous dilution. Raw data exports and concentration calculations were performed using Gen5 software (Winooski, VT), and the quality controls using SAS Software 9.4 (Cary, NC).

*Sample size calculation*

Sample sizes of 63 cases and 63 controls were required to achieve 80% power to detect a difference of 0.2 mean log antibody titer. The calculation was based on a two-sided two-sample t-test with a null hypothesis in which both group means are 0.5 assuming unequal standard deviations of 0.3 for controls and 0.5 for cases.
